# *In vitro* characterization and molecular epidemiology of *Cryptococcus* spp. isolates from non-HIV patients in Guangdong, China

**DOI:** 10.3389/fmicb.2023.1295363

**Published:** 2024-01-15

**Authors:** Penglei Wang, Yongming Li, Lei Gao, Xiang Tang, Dandian Zheng, Kuihai Wu, Luxia Wang, Penghao Guo, Feng Ye

**Affiliations:** ^1^State Key Laboratory of Respiratory Disease, National Clinical Research Center for Respiratory Disease, Guangzhou Institute of Respiratory Health, The First Affiliated Hospital of Guangzhou Medical University, National Center for Respiratory Medicine, Guangzhou, China; ^2^Department of Respiratory Medicine, Longgang Central Hospital, Shenzhen, China; ^3^Microscopy Core Facility, Biomedical Research Core Facilities, Westlake University, Hangzhou, China; ^4^Intensive Care Unit, Guangzhou First People’s Hospital, Guangzhou, China; ^5^Department of Hematology Oncology, Jieyang City People's Hospital, Jieyang, China; ^6^Clinical Medicine Laboratory, Foshan City First People's Hospital, Foshan, China; ^7^Clinical Medicine Laboratory, Southern Military Region General Hospital, Guangzhou, China; ^8^Clinical Medicine Laboratory, Sun Yat-sen University First Affiliated Hospital, Guangzhou, China

**Keywords:** *Cryptococcus*, virulence factor, molecular epidemiology, antifungal susceptibility test, genetic evolution analysis

## Abstract

**Background:**

The burden of cryptococcosis in mainland China is enormous. However, the *in vitro* characterization and molecular epidemiology in Guangdong, a key region with a high incidence of fungal infection in China, are not clear.

**Methods:**

From January 1, 2010, to March 31, 2019, clinical strains of *Cryptococcus* were collected from six medical centres in Guangdong. The clinical information and characteristics of the strains were analysed. Furthermore, molecular types were determined.

**Results:**

A total of 84 strains were collected, mostly from male and young or middle-aged adult patients. Pulmonary and cerebral infections (82.1%) were most common. All strains were *Cryptococcus neoformans*, grew well at 37°C and had capsules around their cells. One melanin- and urea- and one melanin+ and urea- variants were found. Although most strains exhibited a low minimum inhibitory concentration (MIC) value for voriconazole (mean: 0.04 μg/mL) and posaconazole (mean: 0.12 μg/mL), the results for these isolates showed a high degree of variation in the MIC values of fluconazole and 5-fluorocytosine, and resistance was observed for 4 out of 6 drugs. A significant proportion of these strains had MIC values near the ECV values, particularly in the case of amphotericin B. The proportion of strains near the clinical breakpoints was as follows: fluconazole: 3.66%; voriconazole: 3.66%; itraconazole: 6.10%; posaconazole: 13.41%; amphotericin B: 84.15%; 5-fluorocytosine: 2.44%. These strains were highly homogeneous and were dominated by the *Grubii* variant (95.2%), VNI (94.0%), α mating (100%), and ST5 (89.3%) genotypes. Other rare types, including ST4, 31, 278, 7, 57 and 106, were also found.

**Conclusion:**

Phenotypically variant and non-wild-type strains were found in Guangdong, and a significant proportion of these strains had MIC values near the ECV values towards the 6 antifungal drugs, and resistance was observed for 4 out of 6 drugs. The molecular type was highly homogeneous but compositionally diverse, with rare types found. Enhanced surveillance of the aetiology and evolution and continuous monitoring of antifungal susceptibility are needed to provide references for decision-making in the health sector and optimization of disease prevention and control.

## Introduction

*Cryptococcus* species are globally prevalent opportunistic pathogenic fungi that mainly threaten immunocompromised populations, with a mortality of ~15% among patients with acquired immunodeficiency syndrome (AIDS) because of cryptococcal meningitis (CM) (Rajasingham et al., 2017). However, recent studies have also revealed that non-AIDS and immunocompetent patients are increasingly at risk (George et al., 2015; O’halloran et al., 2017). Due to the high mortality rate, *Cryptococcus* has been assigned to the critical priority group in the first fungal priority pathogen list released by the WHO in 2022 to guide research, development and public health action (World Health Organization, 2022). China is one of the most highly endemic areas for these fungal infections, but due to the lack of high-quality epidemiology studies and population-based surveillance data, the true burden of cryptococcosis in China is hard to establish (Chen M. et al., 2018; Zhou et al., 2020). Nevertheless, a risk-based estimate study based on a systematic literature review predicted that a total of 65,507 CM cases would occur annually, with an incidence of 4.57 per 100,000 persons per year (Zhou et al., 2020). The burden might have been underestimated given the rapid increase in newly reported cases and inadequate surveillance systems (Chen M. et al., 2018; Zhou et al., 2020; Chifungi, 2022).

Cryptococcosis is an invasive infection caused by *C. neoformans* or *Cryptococcus gattii*. *C. neoformans* has been the most prevalent, while *Cryptococcus gattii* is rare in China (Chaturvedi and Chaturvedi, 2011; May et al., 2016). Different subtypes of *C. neoformans* have varying pathogenic characteristics and are associated with varying clinical outcomes (Beale et al., 2015; Montoya et al., 2021). The *Grubii* variant is prevalent globally (mainly isolated from bird faeces and eucalyptus). Both patients with AIDS and other immunocompromised patients are susceptible (Nascimento et al., 2017). The *gattii* variant, which is more common in tropical and subtropical regions, has also been reported in specific ecological niches, such as eucalyptus. It is more likely to infect immunocompetent hosts and can cross the blood–brain barrier (Litvintseva et al., 2005; Fang et al., 2015). In contrast, the *neoformans* variant has rarely been reported compared with other variants. The predominant *Cryptococcus* group in China has been the *Grubii* variant, VNI genotype, but this variant mostly infects individuals with no underlying disease (Yuchong et al., 2012; Fang et al., 2015), while in other countries, immunocompromised individuals constitute the high-risk groups (Pyrgos et al., 2013; Rajasingham et al., 2017). In addition, the clinical characteristics are usually nonspecific, resulting in a high rate of missed diagnoses, and despite the availability of optimal treatment, the non-AIDS CM population still has a mortality rate of 13.7 to 42.3% (Shih et al., 2000; George et al., 2018; Pasquier et al., 2018). Therefore, the main *Cryptococcus* strains circulating in China have different features compared with those in other countries. However, *in vitro* characterization and molecular epidemiological studies have been scarce.

Molecular epidemiological studies on *Cryptococcus* have previously been carried out in some areas of China (Liang et al., 2014; Dou et al., 2015, 2017; Chen Y. H. et al., 2018; Xiao et al., 2018; Zhang et al., 2022; Zhou et al., 2022). These studies showed that the *C. neoformans Grubii* variant and VNI type were dominant in China. Rare genotypes specific to local areas with differences in susceptible populations have also been reported. The prevalence of *Cryptococcus* is related to the geographic region and climate. Because Guangdong Province is located in the subtropical region, revealing the epidemiological characteristics of *Cryptococcus* will provide insights into the molecular types and disease patterns of cryptococcosis. In this study, we aimed to investigate the *in vitro* characteristics and molecular epidemiology of *Cryptococcus* strains circulating in Guangdong Province, China, to provide crucial information for the development of effective prevention and control strategies specifically tailored to address the challenges of cryptococcosis in this region.

## Methods

### Collection of clinical specimens

Between January 2010 and March 2019, all clinical strains of *Cryptococcus* from six medical centres in Guangdong (Supplementary material) were collected. The samples consisted of deep sputum, lung tissue, pleural fluid, blood, cerebrospinal fluid or urine. Clinical information, including age, sex, symptoms, diagnosis, time of specimen collection, and specimen source and types, was collected. The Ethics Committee of the First Hospital of Guangzhou Medical University approved the study protocol (No. KE-0254/75/211). The clinical specimens were named according to the order of collection from 6 medical centres (Supplementary methods).

### Identification and preservation of clinical strains

Clinical specimens were transferred to Sabouraud dextrose agar under aseptic conditions and incubated at 30°C for 48–72 h to evaluate growth. Single colonies were selected for ink staining analysis and matrix-assisted laser desorption ionization time-of-flight mass spectrometry (MALDI-TOF/MS) identification. Subsequently, colonies were placed in Microbank™ Preservation Tube Medium and stored frozen at −80°C. Strains were then repurified and identified by performing ink staining and mass spectrometry prior to conducting experiments.

### Detection of virulence factors

Melanin and urease production by cryptococcal strains was examined on melanin agar medium and urea agar medium, respectively. The corresponding agar medium formulations have been detailed in the Supplementary methods section. Single colonies were selected from Sabouraud dextrose agar (after recovery from −80°C) and inoculated into the above media (melanin agar medium and urea agar medium) at 30°C for 3 to 10 days, after which the pigmentation of fungal colonies and the colour of the medium were assessed. The presence of black colonies within 10 days was considered positive for the melanin test, and a change in the colour of the medium to pink or peach was deemed positive for the urease test.

The growth test was carried out on yeast extract peptone dextrose medium at 37°C for 3–7 days and was deemed positive if colonies grew within 7 days. Capsule detection was performed using ink staining for direct microscopic examination. If fungal cells and thick capsules were visible during India ink staining, the test was considered positive for the presence of capsules. *Cryptococcus H99* and *Candida albicans ATCC 76615: 09* served as positive and negative controls, respectively, as previously reported (Bian et al., 2015; Nyazika et al., 2016).

### Antifungal susceptibility testing

The susceptibility of the isolates to fluconazole, voriconazole, itraconazole, posaconazole, amphotericin B, and 5-fluorocytosine was determined using a broth microdilution method. The results were interpreted according to the recommendations of the Clinical and Laboratory Standards Institute. The epidemiologic cut-off values (ECVs) proposed by the previous study (Fan et al., 2016) were used to differentiate wild-type (WT) and non-wild-type (NWT) strains. WT isolates were identified as those for which the MIC of any antifungal agent was above the corresponding ECV, indicating that the isolate had acquired resistance to the agent. The standards for ECVs were as follows: fluconazole: 8 μg/mL; voriconazole: 0.12 μg/mL; itraconazole and posaconazole: 0.25 μg/mL; amphotericin B: 1 μg/mL; 5-fluorocytosine: 8 μg/mL. The antifungal sensitivity tests were repeated twice, and any tests with abnormal results were repeated three times. *Candida parapsilosis* ATCC22019 and *Candida krusei* ATCC6258 were used as quality controls according to the CLSI M27 guidelines (CLSI, 2017; Tóth et al., 2019; Li et al., 2022).

### DNA extraction

Genomic DNA was extracted from standard and clinical strains of *Cryptococcus* according to the instructions of the fungal genomic DNA extraction kit (Beijing Solarbio Science & Technology). The extracted DNA was used for multilocus sequence typing (MLST), variant identification, and genotype and mating analyses.

### *Cryptococcus* variant identification

To identify the isolated fungi, the internal transcribed spacer (ITS) of the ribosomal RNA gene was amplified with the ITS1 and ITS4 primers, and the products were sequenced and compared with the sequences of other *Cryptococcus* strains accessible in the GenBank database to obtain the variant types (Katsu et al., 2004). Detailed experimental conditions can be found in the Supplementary methods.

### *Cryptococcus* genotype identification

The genotype of the isolates was determined by amplification of the superoxide dismutase (SOD)1 gene and performing a BLAST comparison against standard strain sequences (WM148 (serotype A, VNI), WM 626 (serotype A, VNII), Bt63 (serotype A, Botswana), WM 628 (serotype D, VNIII) and WM629 (serotype AD, VNIV)) in the GenBank and MLST online databases, with reference to the methods of Chowdhary et al. (2011) and Chen Y. H. et al. (2018). Detailed experimental conditions can be found in the Supplementary methods.

### *Cryptococcus* mating identification

A pair of mating-specific primers (Chaturvedi et al., 2000) was used for polymerase chain reaction (PCR) amplification to obtain a target band of ~100 bp, and the mating results were achieved by BLAST comparison with the reference strain [mating reference strains: KN99 (Aa) and H99 (Aα)]. Detailed experimental conditions can be found in the Supplementary methods.

### *Cryptococcus* MLST identification

The seven housekeeping genes capsule polysaccharide (CAP59), glycerol-3-phosphate dehydrogenase (GPD1), laccase (LAC1), phospholipase B1 (PLB1), superoxide dismutase (SOD1), uracil phosphoribosyltransferase (URA5), and intergenic spacer region (IGS1) of *Cryptococcus* were amplified by PCR amplification (Meyer et al., 2009). The obtained sequences were aligned with the standard sequences in the MLST database using BioEdit software. Nonrelevant fragments were removed, resulting in corresponding fragments for the seven loci. These fragments were then input into the *Cryptococcus* MLST database^1^ for online comparison, enabling the identification of the corresponding sequence types (STs). Detailed experimental conditions can be found in the Supplementary methods.

### MLST phylogenetic tree analysis

BioEdit software was used to concatenate the sequences of the seven housekeeping genes for clinical isolates (one strain per ST) and reference strains (one strain per different ST and genotype in the MLST database). The concatenated sequences from all strains were compiled. The resulting sequence library was subjected to 1,000 repetitions of the neighbour-joining (N-J) method using MEGA 7.0 software to generate a phylogenetic tree. Detailed experimental conditions can be found in the Supplementary methods.

### Statistical analysis

Data were analysed with SPSS (IBM SPSS Statistics 12.0) statistical software. The enumeration data are shown as the count (percentage), and the continuous data are presented as the mean (standard deviation). The MIC50 and MIC90 of antifungal drugs against isolates were calculated separately by using WHONE software. The percentages of WT and NWT were calculated according to the ECV as defined by the previous study (Fan et al., 2016). The molecular type characteristics of these isolates are expressed as counts (percentages). Trends of Antimicrobial Resistance (AMR) pattern and virulence analysis were performed using linear regression.

## Results

### Patient characteristics

A total of 84 strains were collected, all of which were *C. neoformans,* isolated predominantly from males (*n* = 63, 75.0%) and young or middle-aged patients (*n* = 73, 86.9%). The most frequent specimen sources were cerebrospinal fluid (*n* = 43, 51.2%) and blood (*n* = 18, 21.4%). Two strains were isolated from urine (Table 1). The majority of these patients were diagnosed with cryptococcal meningitis (CM) (*n* = 39, 46.4%), pulmonary cryptococcosis (PC) (*n* = 21, 25%), and haematogenously disseminated cryptococcosis (*n* = 13, 15.5%). Patients with multisite disseminated infection accounted for 10.7% of the total. A significant increase in the number of isolates was demonstrated within 2017–2019 compared with 2010–2016.

**Table 1:** Characteristics of the 84 strains.

Characteristic	All Patients (*N* = 84)
Age	
Median (IQR) — yr	37.0 (30.0–58.0)
Distribution —no. (%)	
≤18	1 (1.2%)
19–35	26 (31%)
36–45	19 (22.6%)
46–55	18 (21.4%)
56–65	10 (11.9%)
>65	10 (11.9%)
Female sex —no. (%)	21 (25.0%)
Specimen source	
Bronchoalveolar lavage fluid	5 (6.0%)
Deep sputum	8 (9.5%)
Lung tissue	7 (8.3%)
Pleural effusion	1 (1.2%)
Blood	18 (21.4%)
Cerebrospinal fluid	43 (51.2%)
Urine	2 (2.4%)
Patients’ diagnosis	
CM	39 (46.4%)
PC	21 (25.0)
Hematogenously disseminated cryptococcosis	13 (15.5%)
PC + CM	2 (2.4%)
Hematogenously disseminated cryptococcosis+CM	6 (7.1%)
Hematogenously disseminated cryptococcosis+PC	1 (1.2%)
Urinary tract infections	2 (2.4%)

IQR, interquartile range; CM, Cryptococcal meningitis; PC, Pulmonary cryptococcosis.

### Cryptococcal virulence factors

**Figure 1:**
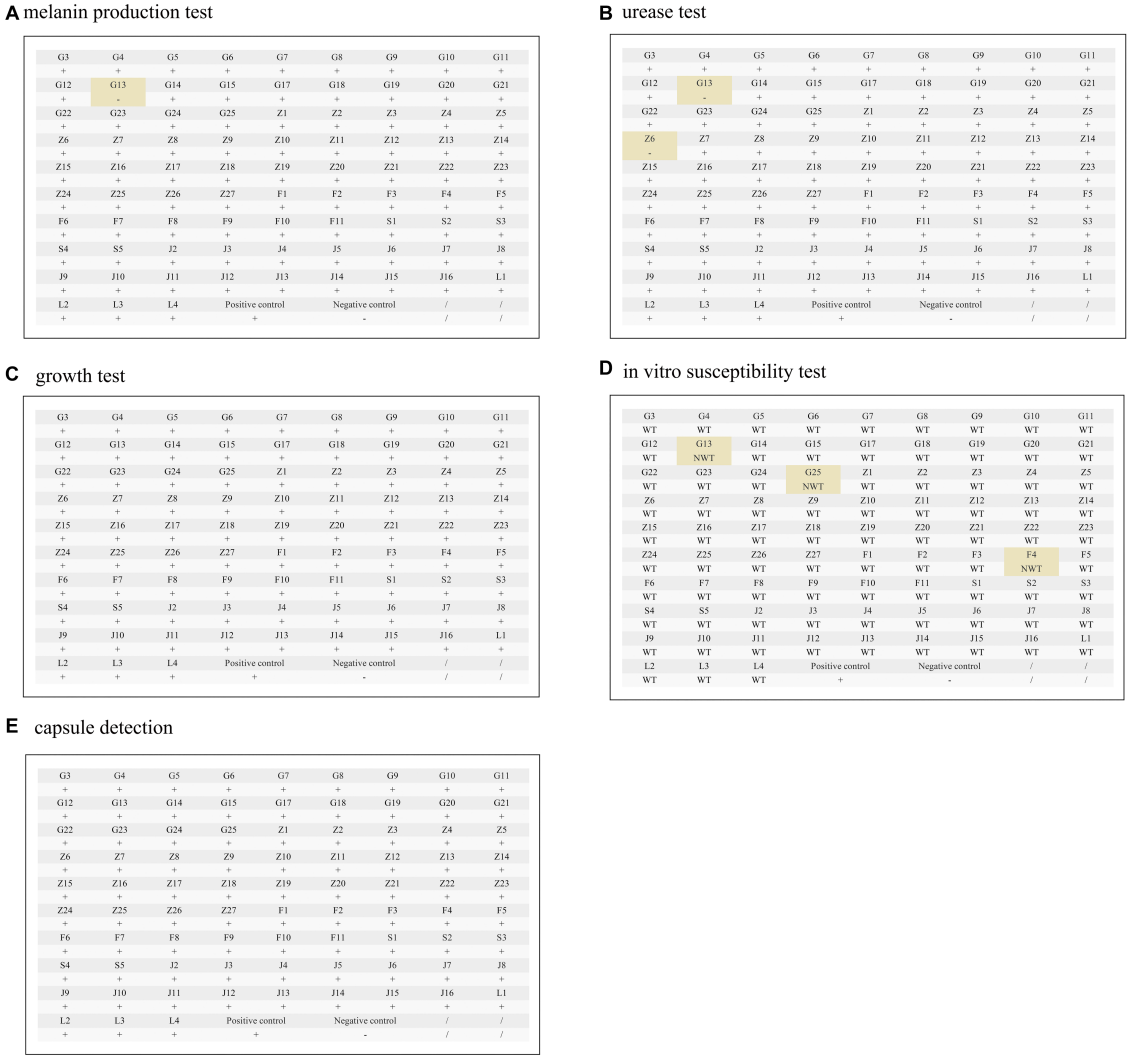
Virulence phenotyping and antifungal resistance of the isolates. **(A)** Melanin production test, **(B)** Urease test, **(C)** Growth test, **(D)** *In vitro* susceptibility test, **(E)** Capsule detection. +: Positive, −: negative; WT: wild type, NWT: non-wild type. The “+” symbol represents a positive result, indicating a drug-resistant strain/a melanin-producing strain/a urease-producing strain/growth at 37°C/the presence of a capsule. The alphanumeric combinations represent the unique identification numbers assigned to each clinical isolate. Further details on specific naming conventions can be found in the supplementary information. *In vitro antifungal susceptibility assays, Z6 and J12 had poor growth in multiple tests and were not included in the statistics. Positive and negative controls are described in the Methods.

All clinical isolates except for strain G13 presented with dark brown colonies when cultured on substrate producing melanin for 10 days. On extended observation for 20 days, only yellowish-white colonies were found in G13. After 48 h of incubation in urea agar medium, the colour of the medium of strains G13 and Z6 did not change, while the medium of the other isolates became reddish. The observation was extended for 20 days, and the results did not change. All isolates were able to form colonies visible to the naked eye after 24–72 h of incubation at 37°C. Fungal cells and thick capsules were visible in all isolates using India ink staining. In all experiments, the results for both the positive and negative controls were in line with expectations (Figure 1). In addition, we examined the temporal trends of phenotypic variations in virulent strains. However, our findings suggest that the discovery of variant strains does not appear to be significantly associated with the year of isolation (Supplementary Figure S1).

### *In vitro* antifungal susceptibility

**Figure 2:**
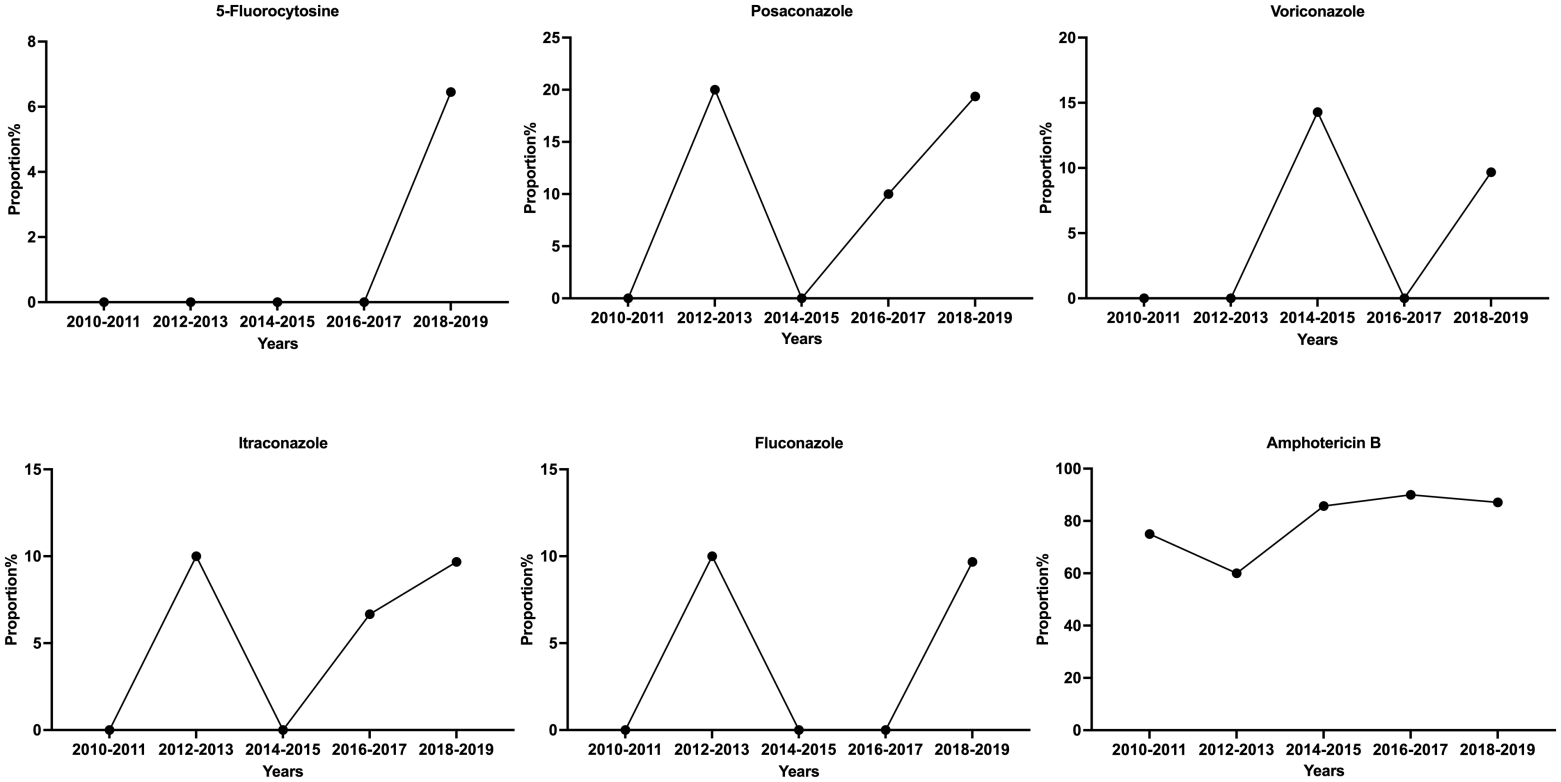
Proportion of strains with MIC values near the ECV values.

Among the 84 isolates for the *in vitro* antifungal susceptibility assays, Z6 and J12 had poor growth in multiple tests and were not included in the analysis. The results for the remaining 82 isolates showed a large degree of variation in the MIC values of fluconazole and 5-fluorocytosine, which were the first-line antifungals. The maximum MICs of 5-fluorocytosine and fluconazole were 64 times higher than the minimum, corresponding to standard deviations of 1.40 and 2.13 μg/mL, respectively. However, the distribution of the MIC values of posaconazole, voriconazole and itraconazole was relatively narrow and maintained in the lower range. Based on the ECV standards of the previous study (Fan et al., 2016), 79 of the 82 strains (96.3%) were sensitive WT strains, while 3 were potentially NWT strains with acquired resistance. Of the three NWT strains, F4 had an MIC of 8 μg/mL for 5-fluorocytosine and fluconazole. In addition, the MICs of the G25 strain for 5-fluorocytosine, itraconazole and fluconazole were consistently higher than the ECVs. In particular, the MIC of fluconazole reached 16 μg/mL. Both strains belonged to the normal phenotype, while G13 was a phenotypic variant NWT strain (urease -, melanin -), exhibiting a relatively low MIC for all agents but an MIC higher than the ECV for amphotericin B. Similarly, another phenotypic variant, Z6 (urease -), was found to be sensitive to almost all the drugs (Figure 1D; Table 2). Although the number of isolated NWT strains was relatively low in different years, we observed that a significant proportion of these strains had MIC values near the ECV values, particularly in the case of amphotericin B. The proportion of strains near the clinical breakpoints was as follows: fluconazole: 3.66%; voriconazole: 3.66%; itraconazole: 6.10%; posaconazole: 13.41%; amphotericin B: 84.15%; 5-fluorocytosine: 2.44% (Figure 2; Supplementary Figure S2).

**Table 2:** *In vitro* susceptibility test of isolated strains.

	5-Fluorocytosine	Posaconazole	Voriconazole	Itraconazole	Fluconazole	Amphotericin B
ECV (ug/mL)*	8	0.25	0.12	0.25	8	1
**All isolates**						
MIC range (ug/mL)	0.125–8.0	0.015–0.25	0.03–0.25	0.015–0.5	0.25–16.0	0.5–2.0
Weighted means (ug/mL)	2.44	0.12	0.04	0.10	2.38	0.94
Standard deviation	1.40	0.06	0.03	0.07	2.13	0.21
MIC 50 (ug/mL)	2.0	0.12	0.03	0.12	2.0	1.0
MIC 90 (ug/mL)	4.0	0.25	0.06	0.12	4.0	1.0
NWT- no. (%)	0(0%)	0(0%)	1(1.22%)	1(1.22%)	1(1.22%)	1(1.22%)
**Phenotypic variant and NWT strains**						
G13	0.125	0.03	0.03	0.03	0.25	2^#^
Z6	<0.12	<0.03	<0.03	<0.03	<0.12	<0.03
G25	8^#^	0.12	0.12	0.5^#^	16^#^	1
F4	8^#^	0.12	0.25	0.12	8	1

ECV, Epidemiologic cut-off values; MIC, Minimal Inhibitory Concentrations; NWT, non-wide-type. *it was according to the previous study (Fan et al., 2016) guidelines. A: G13 had both urease and laccase negative phenotypes while Z6 was a urease negative strain. #: abnormal MIC values.

### Trends of AMR pattern and virulence analysis

During the period from 2010 to 2019, we observed little significant changes in the prevalence of variant strains exhibiting altered phenotypes in virulence factors among the isolates of Guangdong (Supplementary Figure S1). The majority of strains displayed normal virulence phenotypes, surviving at 37°C and exhibiting urease, melanin and capsule production. However, we did identify one strain with a mutation in melanin production during 2016–2017, as well as two strains with variations in urease production between 2013–2014 and 2016-2017.While we did not initially identify any drug-resistant strains in the earlier years, we have observed the emergence of drug-resistant strains between 2016 and 2019. Additionally, there appears to be no significant trends in the proportion of strains near the clinical breakpoints (Figure 2; Supplementary Figure S1).

### Cryptococcal genotypes

Of the amplification products of the ITS of the ribosomal RNA gene, SOD1, mating type and housekeeping gene were used to evaluate the variants, genotypes and corresponding ST. The molecular types in the region were highly homogeneous and were generally dominated by the *Grubii* variant (*n* = 80, 95.2%), VNI genotype (*n* = 79, 94.0%), α mating type (*n* = 84, 100%), and ST5 type (*n* = 75, 89.3%) (Table 3). However, the typing composition was rich, with VNI in the *Grubii* variant, VNIV in the *neoformans* variant, and VGI and VGII genotypes in the *gattii* variant. Genotypically, the majority were VNI (*n* = 79, 80.6%), while other types of VNIV (*n* = 1), VGI (*n* = 3), and VGII (*n* = 1) were also found. Similarly, MLST typing also showed substantial diversity. In addition to the most common ST5 (*n* = 75, 89.3%) and ST31 types (*n* = 2, 2.4%), ST4, ST57 and ST106, which have been reported in other regions of China, were also found (Table 3). In addition, we identified the ST7 and ST278 strains for the first time in China.

**Table 3:** Molecular types of clinical strains of *Cryptococcus neoformans* in Guangdong.

Variant	Genotype	ST type	Mating	No. (%)
***Grubii***				79 (94.0%)
		ST5	α	75 (89.3%)
	VNI	ST31	α	2 (2.4%)
		ST4	α	2 (2.4%)
***Gattii***				4 (4.8%)
	VGI	ST57	α	2 (2.4%)
		ST106	α	1 (1.2%)
				1 (1.2%)
	VGII	ST7	α	1 (1.2%)
				
***Neoformans***				1 (1.2%)
	VNIV	ST278	α	1 (1.2%)

### Phylogenetic tree analysis

**Figure 3:**
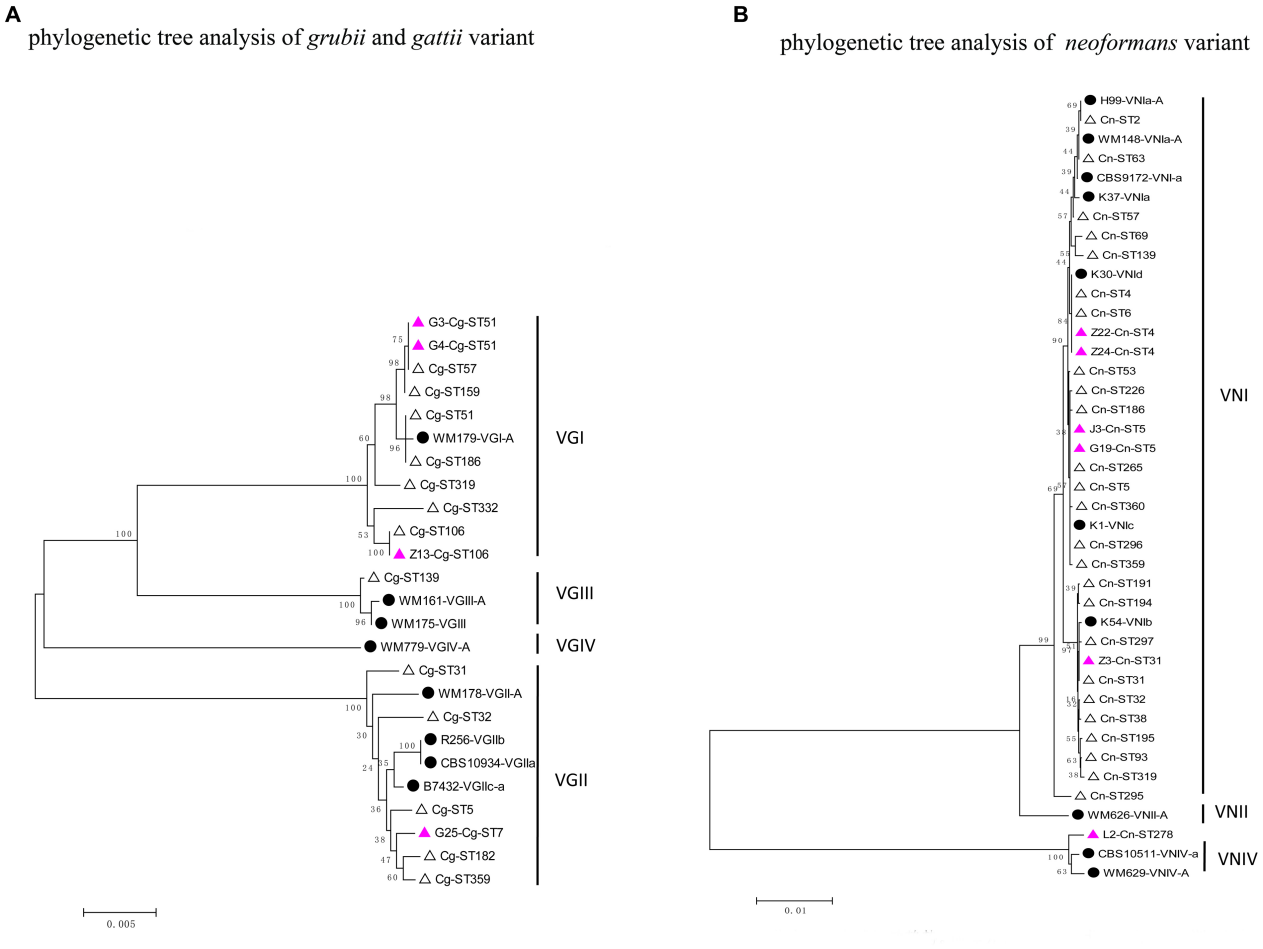
Phylogenetic tree analysis of the isolates based on MLST types. **(A)** Phylogenetic tree analysis of *gattii* and g*rubii* variants. **(B)** Phylogenetic tree analysis of the *Neoformans* variant. The solid black circles represent standard strains of *Cryptococcus*, the hollow triangles represent strains already reported in China, and the purple triangles represent strains found in Guangdong Province.

**Figure 4:**
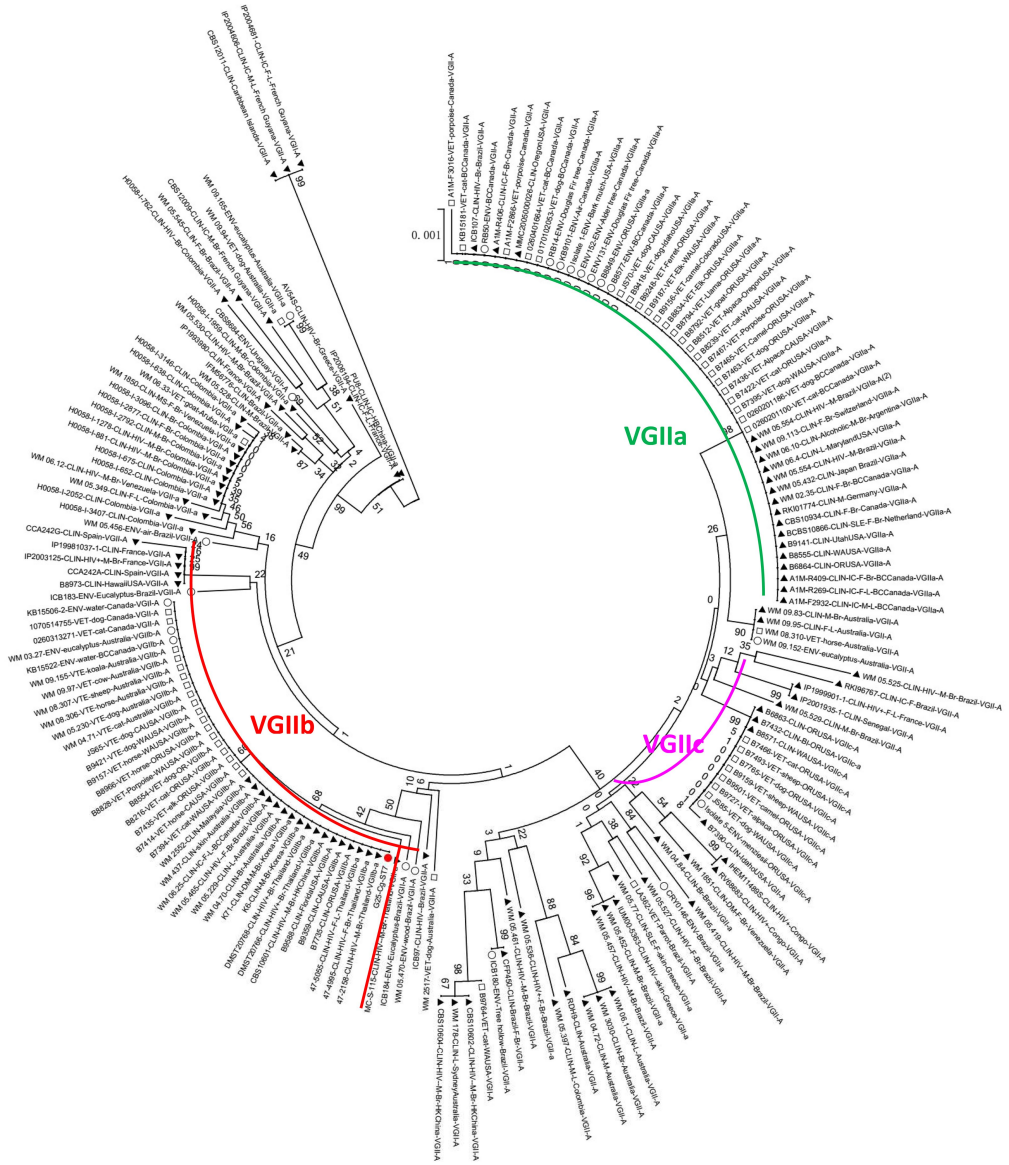
Phylogenetic tree analysis of the VGIIb (ST7) strain. The red circle represents the G25-Cg-ST7-VGIIb strain found in this study, the black triangle represents the clinical strain, the square represents the animal strain, and the hollow circle represents the environmental strain.

The phylogenetic tree was constructed using the neighbour-joining method based on the reported MLSTs in China and the isolates in our study (*Grubii* and *Gattii* variant). Type ST57 and ST16 in Guangdong clustered together, while ST106 was more closely related to type ST319 (Figure 3A). Since the newly discovered type ST7 in Guangdong was distantly related to the other reported STs in China, another phylogenetic tree of the VGII cluster for strain G25-Cg-ST7 was next established. We found that this strain was closely related to the weakly pathogenic VGIIb subtype endemic in North America, Australia, and Southeast Asia (Figure 4). The phylogenetic tree analysis for the *neoformans* variant (Figure 3B) showed that types ST5, ST4 and ST31 in Guangdong clustered with a variety of domestic STs, while ST278 was also identified and was significantly distant from the other reported ST relatives isolated in China.

## Discussion

This study aimed to examine the *in vitro* characteristics and molecular epidemiology of clinical *Cryptococcus* strains from non-HIV patients in Guangdong. Based on the data from six tertiary hospitals in Guangdong, we found for the first time that phenotypically variant and non-wild-type strains were found in Guangdong, and a significant proportion of these strains had MIC values near the ECV values for 6 antifungal drugs, and resistance was observed for 4 out of 6 drugs. Thus, although fluconazole and amphotericin B could remain the first-line agents, continuous monitoring of antifungal susceptibility should be performed due to the potential high risk of resistance. The molecular type was highly homogeneous but compositionally diverse, with rare types found, among which we identified the ST7 and ST278 strains for the first time in China. This could be related to exotic imports, and the virulence of these alien species might be altered in new environments. Enhanced surveillance of aetiology and evolution is needed to provide references for decision-making in the health sector and optimization of disease prevention and control. Our study is the first multicentre pathogenetic and molecular epidemiological study of *Cryptococcus* clinical strains in Guangdong and may have clinical significance in defining the pathogenic distribution and disease patterns of cryptococcosis in this region, as well as for developing health care policies.

Two phenotypic variant strains were found. Z6 was a urease-negative strain, and G13 had both urease- and laccase-negative phenotypes (Figures 1A,B). Little significant changes were observed in the prevalence of variant strains from 2010–2019, this could be related to limited sample size. Since the majority of *Cryptococcus* strains exhibited normal phenotypes, this suggests that future research would benefit from a larger sample size to obtain more conclusive results. Besides, We also found that, apart from amphotericin B, these two phenotypic variant strains had abnormally low MICs (G13) and poor growth (Z6), suggesting that the phenotypic changes may have led to lower *in vitro* resistance than that of the phenotypically normal strains (Table 2). The G13 host showed significant signs of meningeal irritation and intracranial hypertension before treatment, whereas Z6 was derived from a 37-year-old non-AIDS patient with CM without significant underlying disease. Therefore, both strains of phenotypic variants had significant intracranial invasiveness. The presence of a virulence factor may be a manifestation of the resistance against the habitat (Levitz, 1999; Sabiiti et al., 2014; Moreno et al., 2017; Janbon, 2018), rather than solely an indicator of infection and disease in hosts. The relationships among virulence factors, environmental tolerance, and pathogenicity deserve more attention.

The majority of *Cryptococcus* strains in this region demonstrated varying degrees of susceptibility to common antifungal agents, which was generally consistent with reports from other regions in China (Liang et al., 2014; Bian et al., 2015; Dou et al., 2015, 2017; Chen Y. H. et al., 2018). However, some non-wild-type strains that were not susceptible to fluconazole, itraconazole, fluorocytosine and amphotericin B and strains with MICs near the clinical breakpoints were also found (Table 2; Figure 2). In particular, the G25 strain had a high MIC of 16 μg/mL for fluconazole. Although the number of isolated NWT strains was relatively low in different years, a significant proportion of these strains had MIC values near the ECV values, which may indicate reduced susceptibility to the drugs. These findings are similar to those of other studies in China (Chen et al., 2008; Dou et al., 2015; Fan et al., 2016; Yan-Hui et al., 2018). A notable increase in the proportion of fluconazole-susceptible non-wild-type strains was observed, and the fluconazole susceptibility and nonsusceptibility rates (intermediate and resistant) were 73.7 and 26.3%, respectively, for *Cryptococcus* during 2009 and 2014 (Fan et al., 2016; Xiao et al., 2018). Similarly, strains with 5-fluorocytosine and fluconazole MIC >16 μg/mL have also been reported in other Asian countries, such as Thailand, India and Indonesia (Khayhan et al., 2013). To date, the rate of resistance to amphotericin B ranges from 1 to 75% (Li et al., 2020; Zhang et al., 2022; Zhou et al., 2022). While there is no apparent upward trend in the annual increase of antifungal resistance among *Cryptococcus* strains in the Guangdong region, a substantial proportion of isolates do exhibit MIC values near the clinical breakpoints, particularly against amphotericin B. This finding emphasizes the critical importance of resistance surveillance and it is imperative to conduct future research studies with a larger sample size to gain a more comprehensive understanding and validate this trend.

Several reports might attribute this disseminated MIC elevation to the long course and high frequency of clinical dosing (Denning, 2003; Kontoyiannis and Lewis, 2014). However, this conclusion deserves more investigation. Prolonged *in vitro* fungal drug coculture can induce the development of drug-resistant strains, and theoretically, this could also occur in patients with a prolonged course of dosing. However, we found that the non-wild type and several other strains with a low susceptibility to itraconazole in this study were isolated prior to the initiation of treatment or in the period of empirical antifungal therapy within 2 weeks, and hence, there was no evidence of long-term medication dosing. Similarly, the clinical strains in Thailand and Indonesia showed higher MICs for 5-fluorocytosine, which was not widely used in the two countries (Khayhan et al., 2013). Only 36.4% of these non-fluconazole-susceptible strains were treated with fluconazole in China (Fan et al., 2016). Therefore, there might be other factors that contributed to the acquired antifungal resistance in *Cryptococcus*, and the environmental stress in the habitat was also likely to have played a role. Therefore, a comprehensive analysis in the context of clinical use and prognosis in the region should be carefully performed. In addition, studies have also found that the emergence of drug-resistant strains could be associated with gene mutations (Li et al., 2020; Zhang et al., 2022; Zhou et al., 2022). Notably, although itraconazole, voriconazole and posaconazole are not recommended as the first choice by the guidelines for the treatment of cryptococcosis, clinical strains in the region maintain a low MIC (Table 2), which was consistent with the findings from other studies (Khayhan et al., 2013; Fan et al., 2016) that indicated that itraconazole, voriconazole and posaconazole can be used as alternatives when the strains isolated from patients became resistant to first-line agents.

Significant genetic homogeneity in the molecular types of these clinical isolates in Guangdong was observed, with a predominant population infected with the *Grubii* variant, VNI genotype, α mating type and ST5 type (Table 3). These findings were consistent with those reported from other regions in China (Xiao-Bo et al., 2008; Bian et al., 2015; Dou et al., 2015) but different from those reported in Brazil, Australia and the United States (Byrnes et al., 2009; Mora et al., 2010; Carriconde et al., 2011). Rare subtypes such as *Neoformans* variants, rare genotypes (VNIV, VGI, VGII) and rare STs (ST4, 7, 57, 106, 278) were also found, representing the diversity of composition. In our study, four genotypes (VNI, VNIV, VGI and VGII) were isolated from Guangdong. Further phylogenetic analysis revealed that most of the VNI genotypes clustered with the VNIc subgroup, consistent with another report from China (Chen et al., 2008), reflecting a high degree of genetic homogeneity. In addition, the VNIV genotype, a very rare *Neoformans* variant, was identified; phylogenetic analysis indicated that this genotype originated in Europe (Figure 3B), suggesting the existence of import pathways for foreign populations and species. Notably, the VG II genotype of a *gattii* variant was identified as VGIIb (ST7); clinically, this genotype exhibited intracranial invasiveness. Phylogenetically, this strain was related to the low-endemicity, weakly pathogenic subgroup VGIIb (ST7) (R272) on Vancouver Island (Figures 3, 4) (Carriconde et al., 2011; Liang et al., 2014). However, unlike the weakly pathogenic, low-virulence characteristics of the North American strains, the strains in Guangdong appeared to be more aggressive in nature. There has been significant genetic recombination behavior and strong mating ability observed among VGII genotype strains (Campbell et al., 2005; Chen et al., 2008; Liang et al., 2014), which can lead to virulence variation and environmental adaptability. Therefore, these results indicated that the diversity of molecular types in Guangdong could be related to exotic imports and that the pathogenicity and some features of those alien species might be altered. Enhanced surveillance of exotic importation is needed.

Although ST5 and ST31 dominated (Table 3), some rare types were also found, which differed from previous studies that reported a single and closed population composition in Guangdong (Peng-Hao et al., 2016). This indicates that the population composition has diversified recently, possibly as a result of phenotypic or even genotypic mutations in response to the environment, internal reorganization, and the import of exotic populations. Furthermore, ST7 formed its own group (Figure 3A), while ST278 was more distantly related to the STs that have already been reported in China (Figure 3B) and were mainly distributed in Europe. Therefore, dynamic monitoring of the STs of *Cryptococcus* in different geographical settings can help to clarify the population composition of pathogens and the migration of exotic populations. Our data were consistent with those from other regions in China and other countries (Fan et al., 2016), mostly with the MATα mating and the lack of the a-mating type. Reports of a-mating type strains in other parts of China have been rare (Feng et al., 2008; Fan et al., 2016). The MATα strains were more virulent and could penetrate the blood–brain barrier more easily than MAT a (Barchiesi et al., 2005; Nielsen et al., 2005). However, whether this skewed distribution in Guangdong Province is related to virulence or a dominant distribution needs further study.

The main limitation was that all strains were isolated from clinical samples, without the inclusion of environmental strains. Thus, the significance of environmental strains in revealing the transmission routes of *Cryptococcus* and disease prevention and control has not been adequately studied. In addition, we did not perform gene mutation analysis of these strains due to the susceptibility of cryptococcosis to first-line drugs in this area. However, the epidemiological findings of our study can still help to determine the pathogenic distribution and disease pattern of cryptococcosis and provide a reference for the management of *Cryptococcus* in Guangdong.

In conclusion, our study showed that phenotypically variant and non-wild-type strains were found in Guangdong, and a significant proportion of these strains had MIC values near the ECV values for the 6 antifungal drugs, and resistance was observed for 4 out of 6 drugs. The molecular type was highly homogeneous but compositionally diverse, with rare types found. This could be related to exotic imports, and the pathogenicity of those alien species might be altered in new environments. Enhanced surveillance of the aetiology and evolution and continuous monitoring of antifungal susceptibility are needed to provide references for decision-making in the health sector and optimization of disease prevention and control. These findings provide a reference for the management of *Cryptococcus* in Guangdong and other areas, which deserves further investigation.

## Data availability statement

The original contributions presented in the study are included in the article/Supplementary material, further inquiries can be directed to the corresponding author.

## Ethics statement

The studies involving humans were approved by the Ethics Committee of the First Hospital of Guangzhou Medical University approved the study protocol (No. KE-0254/75/211). The studies were conducted in accordance with the local legislation and institutional requirements. The participants provided their written informed consent to participate in this study.

## Author contributions

PW: Conceptualization, Data curation, Formal analysis, Investigation, Methodology, Project administration, Software, Visualization, Writing – original draft, Writing – review & editing. YL: Conceptualization, Data curation, Formal analysis, Investigation, Methodology, Project administration, Software, Visualization, Writing – original draft, Writing – review & editing. LG: Formal analysis, Methodology, Software, Visualization, Writing – original draft. XT: Data curation, Investigation, Resources, Writing – original draft. DZ: Data curation, Investigation, Resources, Writing – original draft. KW: Data curation, Investigation, Resources, Writing – original draft. LW: Data curation, Investigation, Resources, Writing – original draft. PG: Data curation, Investigation, Resources, Writing – original draft. FY: Formal analysis, Funding acquisition, Investigation, Project administration, Resources, Supervision, Validation, Writing – original draft, Writing – review & editing.

## Footnotes

^1^ http://mlst.mycologylab.com
