# Supplementary material for: In vitro characterization and molecular epidemiology of Cryptococcus spp. isolates from non-HIV patients in Guangdong, China
Source: Front Microbiol. 2024 Jan 15;14:1295363. doi: 10.3389/fmicb.2023.1295363 (PMC10823435; doi:10.3389/fmicb.2023.1295363)
Supplement: Supplementary file 1 [file Data_Sheet_1.docx]

**Supplementary Materials**

**Methods:**

**Six medical centers in Guangdong**

1. The first affiliated Hospital of Guangzhou Medical University (strains collected from this hospital were named **“G+number”**)
2. The first affiliated Hospital of Sun Yat-sen University (strains collected from this hospital were named **“Z+number”**)
3. The first people's Hospital of Foshan City (strains collected from this hospital were named **“F+number”**)
4. The eighth people's Hospital of Guangzhou (strains collected from this hospital were named **“S+number”**)
5. General Hospital of Southern Theatre Command (strains collected from this hospital were named **“L+number”**)
6. The people's Hospital of Jieyang City (strains collected from this hospital were named **“J+number”**)

**Table 1:Melanin agar medium formulations**

| Components | Quality | Formula |
| --- | --- | --- |
| KH2PO4 | 4.0g | 1.Dissolve 15g of agar in 100ml of distilled water. Autoclave at 121°C for 15 minutes for high-pressure sterilization.  2.Add the remaining ingredients to 900ml of distilled water. Stir thoroughly until dissolved. Filter through a sterile membrane.  3.Cool the agar solution to around 50°C and add the filtered solution prepared in step 2. Mix well and pour into Petri dishes within 20 minutes.  4.After solidification, wrap the plates with cling film and store at 4°C for future use. |
| MgSO4·7H20 | 2.5g |  |
| Vitamin B1 | 10.0mg |  |
| biotin(Vitamin H) | 20.0ug |  |
| Glucose | 5.0g |  |
| L-asparagine | 1.0g |  |
| DOPA | 200.0mg |  |
| Agar | 15.0g |  |
| Distilled water | 1000.0ml |  |

**Table 2:Urea agar medium formulations**

| Components | Quality | Formula |
| --- | --- | --- |
| Glucose | 1.0 g | 1Dissolve peptone, glucose, NaCl, KH2PO4, and urea in 100 ml distilled water. Mix well and add 0.012 g of phenol red. Adjust the pH to 6.0±0.1 at room temperature.  2Filter the prepared solution from step 1 to sterilize.  3Add 15 g of agar to 3900 ml of distilled water. Autoclave at high pressure (121°C, 15 minutes).  4Cool the agar solution to 50°C~55°C and aseptically add the liquid from step 2. Mix thoroughly and pour into slanted tubes.  5Store at 4°C for future use. |
| Agar | 15.0 g |  |
| Peptone | 1.0 g |  |
| Sodium chloride | 5.0 g |  |
| KH2PO4 | 2.0 g |  |
| Urea | 20.0 g |  |
| Phenol red | 0.012 g |  |
| Distilled water | 1000.0ml |  |

**PCR condition for Cryptococcus variant identification**

Primers:
Forward primer ITS1: 5'-GGAAGTAAAAGTCGTAACAAGG-3'
Reverse primer ITS4: 5'-TCCTCCGCTTATTGATATGC-3'

**Table 3: PCR reaction system for Cryptococcus variant identification**

| Component | Volume (μL) |
| --- | --- |
| 2X EasyTaq PCR SuperMix (+dye) | 25 |
| DNA (20 ng) | 2 |
| ITS1 (5 μmol/L) | 2 |
| ITS4 (5 μmol/L) | 2 |
| ddH2O | 19 |
| Total | 50 |

PCR Reaction Conditions:

Initial denaturation: 94°C for 5 minutes.

Denaturation: 94°C for 60 seconds.

Annealing: 50°C for 60 seconds.

Extension: 72°C for 60 seconds.

Repeat steps 2-4 for 30 cycles.

Final extension: 72°C for 10 minut**es.**

**PCR condition for Cryptococcus genotype identification**

(1) Primers:

For the Cryptococcus neoformans:

Forward primer CNF: 5'-AAGCCTCTCATCCATATCTT-3'

Reverse primer CNR: 5'-TTCAACCACGAATATGTA-3'

For the Cryptococcus gattii:

Forward primer CGF: 5'-GATCCTCACGCCATTACG-3'

Reverse primer CGR: 5'-GAATGATGCGCTTAGTTGGA-3'

**Table 4: PCR reaction system for Cryptococcus genotype identification**

| Component | Volume (μL) |
| --- | --- |
| 2X EasyTaq PCR SuperMix (+dye) | 25 |
| DNA (20 ng) | 2 |
| CNF/CGF (5 μmol/L) | 2 |
| CNF/CGR (5 μmol/L) | 2 |
| ddH2O | 19 |
| Total | 50 |

PCR Reaction Conditions:

Initial denaturation: 94°C for 3 minutes.

Denaturation: 94°C for 30 seconds.

Annealing: 52°C for 30 seconds.

Extension: 72°C for 1.5 minutes.

Repeat steps 2-4 for 35 cycles.

Final extension: 72°C for 10 minutes.

**PCR condition for Cryptococcus mating identification**

(1) Primers:
For α-mating type specific:
Forward primer α -mating-type-specific F: 5'-CTTCACTGCCATCTTCACCA-3'
Reverse primer α -mating-type-specific R: 5'-GACACAAAGGGTCATGCCA-3'

For A-mating type specific:
Forward primer A- mating-type-specific F: 5'-CGCCTTCACTGCTACCTTCT-3'
Reverse primer A- mating-type-specific R: 5'-AACGCAAGAGTAAGTCGGGC-3'

**Table 5: PCR reaction system for Cryptococcus mating identification**

| Component | Volume (μL) |
| --- | --- |
| DNA (20 ng) | 2 |
| 2X EasyTaq PCR SuperMix (+dye) | 10 |
| α -mating primer F (5 μmol/L) | 1 |
| α -mating primer R (5 μmol/L) | 1 |
| ddH2O | 6 |
| Total | 20 |

PCR Reaction Conditions:

Initial denaturation: 95°C for 3 minutes.

Denaturation: 94°C for 1 minute.

Annealing: 57.5°C for 1 minute.

Extension: 72°C for 1 minute.

Repeat steps 2-4 for 30 cycles.

Final extension: 72°C for 10 minutes.

**Table 6: PCR condition for Cryptococcus MLST identification**

| **Gene** | **Primer (5'-3')** | **PCR Amplification Conditions:** |
| --- | --- | --- |
| CAP59 | F :CTCTACGTCGAGCAAGTCAAG R:TCCGCTGCACAAGTGATACCC | 94°C3min;35cycles: 94°C30s, 56°C30s, 72°C1min |
| GPD1 | F:CCACCGAACCCTTCTAGGATA R:CTTCTTGGCACCTCCCTTGAG | 94°Cmin;35cycles: 94°C45s, 63°C1min, 72°C2min |
| IGS1 | F:ATCCTTTGCAGACGACTTGA R:GTGATCAGTGCATTGCATGA | 94°C3min;35cycles: 94°C30s, 60°C30s, 72°C1min |
| LAC1 | F:AACATGTTCCCTGGGCCTGTG R:ATGAGAATTGAATCGCCTTGT | 94°C3min;30cycles: 94°C30s, 58°C30s, 72°C 1min |
| PLB1 | F:CTTCAGGCGGAGAGAGGTTT30 R:GATTTGGCGTTGGTTTCAGT | 94°C3min;30cycles: 94°C45s, 61°C45s, 72°C1min |
| SOD1 | CNF:AAGCCTCTCATCCATATCTT CNR:TTCAACCACGAATATGTA CGF:GATCCTCACGCCATTACG CGR:GAATGATGCGCTTAGTTGGA | 94°C3min;35cycles: 94°C30s, 52°C30s, 72°C1.5min |
| URA5 | F:ATGTCCTCCCAAGCCCTCGAC R:TTAAGACCTCTGAACACCGTACTC | 94°C3min;35cycles: 94°C45s, 63°C1min， 72°C2min |

**Results**

**
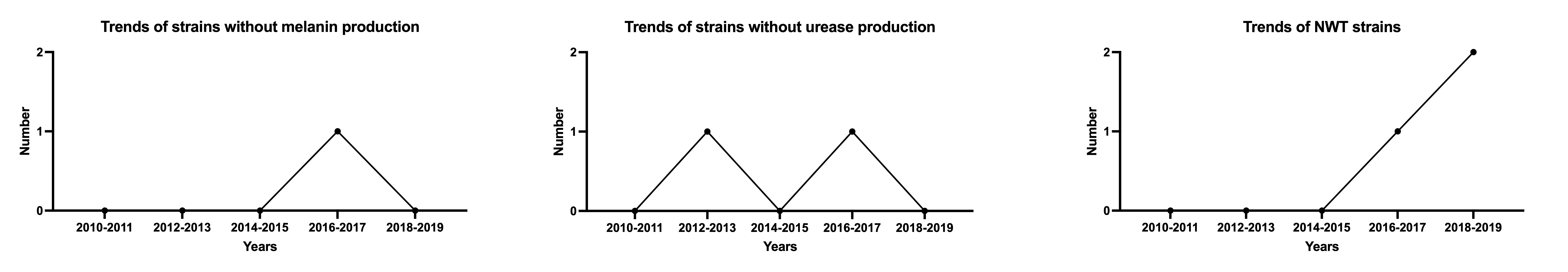
**

**Supplemental figure 1. Trends of phenotypically variant and non-wild-type strains of isolates in Guangdong**

NWT: non-wild-type. Given that all strains exhibit viability at 37℃ and possess capsules, there is no presentation of trend charts for these two variables in this study.

**
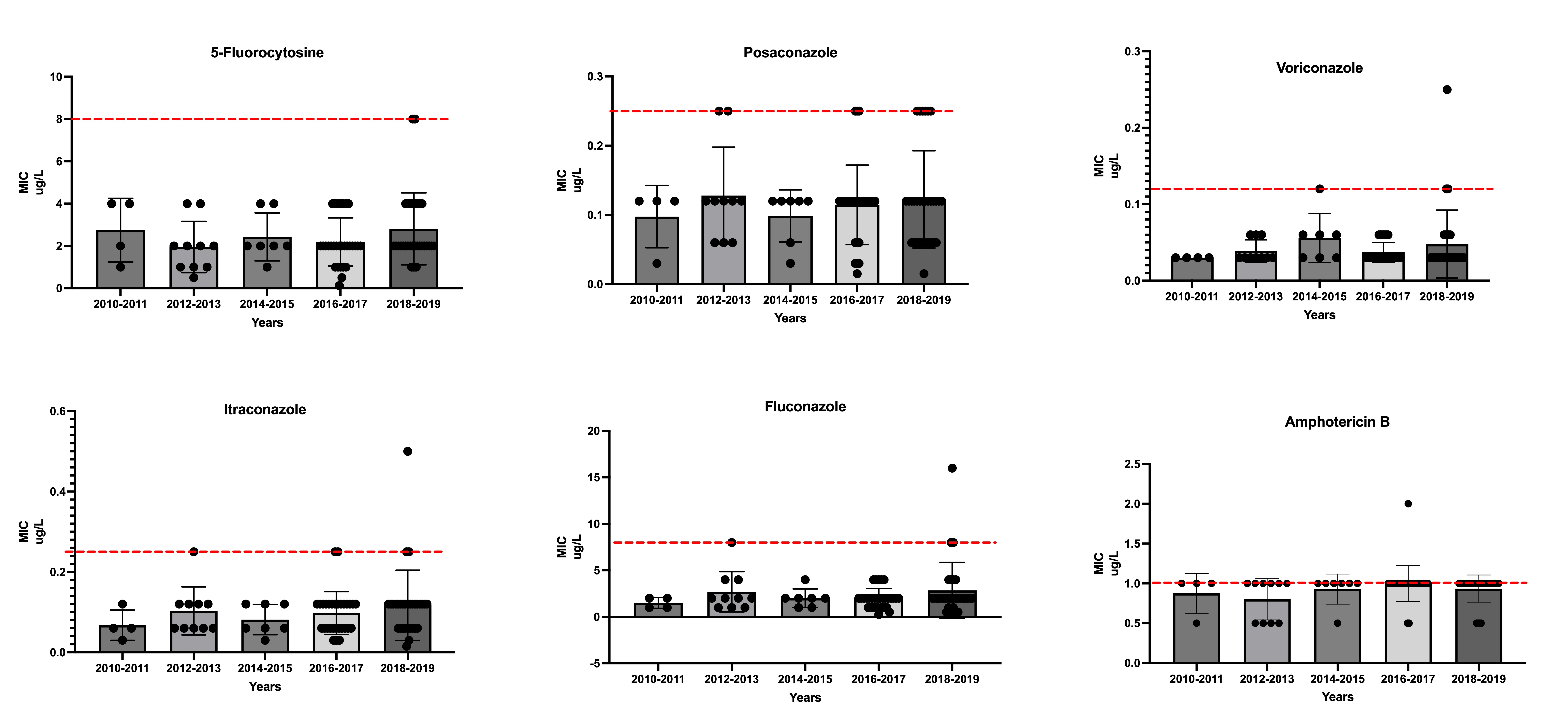
**

**Supplemental Figure 2: Trends of MICs of isolates in Guangdong**

**The red line represents the epidemiologic cut-off (ECV) values.**
